# Hyperendemic Chagas Disease and the Unmet Need for Pacemakers in the Bolivian Chaco

**DOI:** 10.1371/journal.pntd.0002801

**Published:** 2014-06-05

**Authors:** Eva H. Clark, Jackie Sherbuk, Emi Okamoto, Malasa Jois, Gerson Galdos-Cardenas, Julio Vela-Guerra, Gilberto Silvio Menacho-Mendez, Ricardo W. Bozo-Gutierrez, Antonio B. Fernandez, Thomas C. Crawford, Rony Colanzi, Robert H. Gilman, Caryn Bern, for the Working Group on Chagas Disease in Bolivia and Peru

**Affiliations:** 1 University of Alabama at Birmingham, Birmingham, Alabama, United States of America; 2 New York University School of Medicine, New York, New York, United States of America; 3 Rowan University of School of Osteopathic Medicine, Stratford, New Jersey, United States of America; 4 Universidad Catolica San Pablo, Santa Cruz, Bolivia; 5 Salvacor Foundation, Santa Cruz, Bolivia; 6 Centro de Salud Eiti, Gutierrez, Bolivia; 7 Hospital Municipal Camiri, Camiri, Bolivia; 8 Hartford Hospital, Hartford, Connecticut, United States of America; 9 University of Michigan Health System, Ann Arbor, Michigan, United States of America; 10 Johns Hopkins Bloomberg School of Public Health, Baltimore, Maryland, United States of America; 11 University of California San Francisco, San Francisco, California, United States of America; US Food and Drug Administration, United States of America

## Overview

Morbidity and mortality from Chagas cardiomyopathy have declined over the last three decades because of disruption of domestic vector-borne transmission, improved *Trypanosoma cruzi* infection treatment programs, and increasing availability of advanced cardiac care. However, the Gran Chaco, an ecological zone that includes parts of Argentina, Bolivia, and Paraguay, continues to struggle with extremely high rates of vector infestation and *T. cruzi* infection. In addition, this region is one of the poorest in the world, with most individuals living on less than US$2 per day. We estimate that thousands of patients are in need of pacemakers secondary to advanced Chagas cardiomyopathy. However, the vast majority of these individuals lack the resources to obtain these life-saving devices. A collaborative effort must be made by pacemaker donation programs, local implantation hospitals, and the governments of countries affected by Chagas disease to address this unmet need. With the necessary cooperation and infrastructure, pacemaker reuse programs have the potential to offer thousands of low-cost devices to impoverished patients with advancing Chagas cardiomyopathy.

## Introduction

The Southern Cone Initiative to control Chagas disease is a major public health success story [1]. Household insecticide spray programs have greatly diminished infestation by *Triatoma infestans,* the major domestic vector, blood bank and congenital Chagas disease screening have been implemented widely, and the estimated prevalence of *Trypanosoma cruzi* infection has fallen by more than 50% in the last 20 years [2]. Morbidity and mortality from Chagas cardiomyopathy, the most serious manifestation of *T. cruzi* infection, have declined progressively with disruption of domestic vector-borne transmission and increasing availability of advanced cardiac care [3]. The major exception to this pattern of success is the Gran Chaco, an ecological zone shared among Argentina, Bolivia, and Paraguay, and host to the highest rates of vector infestation and *T. cruzi* infection ever reported. Challenges include rapid reinfestation after spray campaigns, insecticide resistance, and sylvatic *Tri. infestans* populations [4]–[6].

Chagas cardiomyopathy occurs in an estimated 20%–30% of *T. cruzi*-infected individuals and features a chronic inflammatory process affecting the conduction system and myocardium [2], [7]. The earliest signs are typically bundle branch blocks and segmental wall motion abnormalities, usually beginning in early adulthood [8]. Later, patients may develop ventricular tachycardia, severe sinus bradycardia, high degree atrioventricular block (AVB), apical aneurysm, progressive dilated cardiomyopathy, and thromboemboli [2]. Syncope from heart block or severe bradycardia is common. Once conduction system abnormalities or arrhythmias are present, patients have shortened survival; signs of left ventricular dysfunction are associated with high short-term mortality [2]. Where accessible, advanced cardiac management has significantly improved the prognosis for patients with Chagas cardiomyopathy [9].

In 2011, we conducted a study of *T. cruzi* infection in seven villages in the Bolivian Chaco [6]. *T. cruzi* infection prevalence was 26.2% among residents younger than 20, 85.4% among 20–29-year-olds and 96.7% among participants 30 years or older. We then offered electrocardiograms (ECGs) to all study participants 20 years or older with *T. cruzi* infection. A total of 327 seropositive participants, 59% of infected study participants, had an ECG evaluated by the study cardiologists. Within a few days, we began to see patients who urgently needed pacemakers. In this article, we present a patient case report and a brief description of other patients found to need pacemakers, and we place these findings in the larger context of the Bolivian Chaco and the impact of Chagas heart disease there.

## Case Report

**Figure 1:**
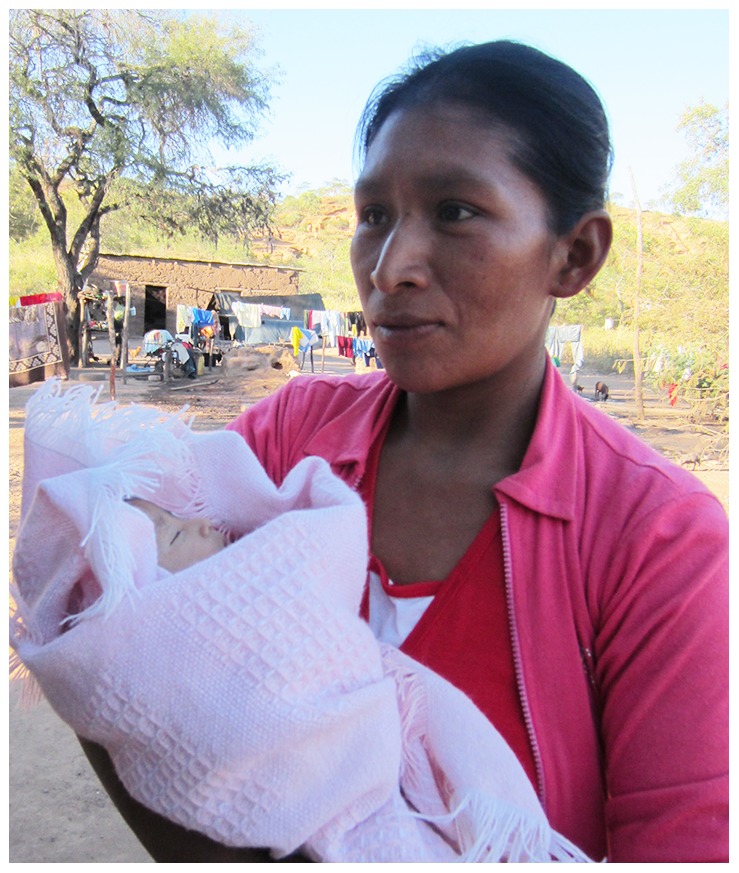
Photograph of BMO and her youngest child.

BMO was a 30-year-old female living in an adobe house with her husband and eight children (Figure 1). She was confirmed to have *T. cruzi* infection during our village study [6]. During her clinical evaluation, she reported fatigue, dizziness, and several episodes of syncope while performing her daily job of washing clothes. Her ECG in October 2011 showed sinus rhythm with complete AVB and a junctional escape rhythm at 43 beats per minute (bpm). Her echocardiogram revealed left ventricular ejection fraction (LVEF) of 40%–45%, interventricular dyssynchrony, mild left atrial dilation, and an enlarged left ventricle (diameter 57.6 mm). Her heart rate was consistently below 50 bpm and failed to increase on exertion. The study cardiologists (ABF, TCC) recommended pacemaker implantation, the costs to be covered by donations. No one in her village had ever had a pacemaker. She and her husband asked our team many questions. Would she be able to care for their eight children and work after surgery? Is there any cure for her illness other than surgery? How would she navigate the complicated medical system? Nearly a year passed before the patient and her family decided to proceed. However, only a few days after beginning presurgical planning, she found that she was pregnant, and the surgery was deferred. She had dyspnea, dizziness, palpitations, and fatigue that worsened throughout her pregnancy. In March 2013, the patient gave birth by uncomplicated Caesarian section to healthy, uninfected twins. Her postpartum ECG again showed complete AVB. Ten days postpartum, she was transported by ambulance to a large public hospital in Santa Cruz. At the time of presentation to the cardiovascular surgeon, she reported one year of dizziness, palpitations, and dyspnea with moderate effort, with intensified symptoms before and just after delivery. Her heart rate was 54 bpm and blood pressure was 117/70. The point of maximal cardiac impulse was displaced to the sixth intercostal space in the anterior axillary line. Her chest X-ray showed a cardiothoracic ratio of 0.54. Complete blood count and metabolic panels were normal. On the first night of hospitalization, her heart rate dropped into the 30 s with transient loss of consciousness. On hospital days 1 and 2, her blood pressure was maintained on dopamine. On day 3, she underwent successful implantation of a dual chamber pacemaker, set at a lower rate limit of 60 bpm. One month after surgery, the patient reported feeling well.

## Discussion

Although migration has brought many infected people to cities in Latin America and beyond, Chagas disease continues to be an affliction of the poor, with rural roots. Poverty and rural housing with earthen walls are consistently associated with higher risk of *T. cruzi* infection and cardiomyopathy [10]. Bolivia remains the poorest country in continental Latin America, with a per capita gross national income (GNI) of US$2,220; 28% of Bolivians live on less than US$2 per day [11]. Our study site is the epitome of a rural population overwhelmed by Chagas disease [6]. Although we lack direct data on income, most residents likely belong to the quarter of Bolivians living on less than US$2 per day; 98% of houses were constructed of either adobe or *barro y palo* (mud-and-sticks).

Data from a representative survey of rural residents in the Gran Chaco province of Tarija Department showed *T. cruzi* prevalence of 71.3% in those 21–49 years old and 87.9% in those 50 or older [12], figures very similar to the prevalence estimated by our survey in Cordillera province, suggesting similarly high prevalence across the six provinces identified by the Bolivian National Chagas Disease Control Program as hyperendemic. These six provinces (Hernando Siles and Luis Calvo of Chuquisaca, Cordillera and Vallegrande of Santa Cruz Department, and Gran Chaco and Burnet O'Connor of Tarija Department) comprised a population of approximately 400,000 in 2010; based on the age structure reported in the Bolivian census, approximately half of these residents were 20 years or older [13]. Based on infection prevalence of 71% in 20–49-year-olds and 88% in those 50 or older, approximately 165,000 Chaco residents older than 20 years are currently infected with *T. cruzi*.

In our study, 12% of those evaluated by ECG had abnormalities characteristic of Chagas disease (predominantly RBBB, LBBB, LAFB, and AV blocks). Eight patients (2.4% of those with ECG data) had an immediate indication for a pacemaker; their mean age was 43 years old (range: 28–58). We used these data and the *T. cruzi* seroprevalence estimated by Chippaux [12] to make an approximate estimate of the need for pacemakers in the Chaco of Bolivia. If the proportion of *T. cruzi*-infected individuals needing a pacemaker is similar to that in our study villages, this would imply that between 3,000 and 4,000 people in the Bolivian Chaco are in need of pacemakers. The inhabitants of the Bolivian Chaco comprise only 7%–10% of the 4–5 million people living in the Gran Chaco [14]; including the Argentine and Paraguayan Chaco, there may be tens of thousands of people in urgent need of pacemakers due to Chagas disease in this zone.

Approximately 40 pacemaker implantations and battery replacements are performed annually in the largest public hospital in Santa Cruz Department (Hospital San Juan de Dios, Department of Statistics, 2012). This hospital serves as the referral center for southern Bolivia, including the Gran Chaco. The vast majority are performed for patients with sufficient resources to pay for the device and surgery (approximately US$5,000, more than twice the per capita GNI of Bolivia), or among the small percentage of patients with private insurance coverage. Many other pacemakers are implanted in private centers in Santa Cruz, but only more affluent patients have access to these services. Clearly, few options are available to impoverished Bolivians, who make up the great majority of Chagas disease patients.

Three reduced-cost pacemaker implantation programs operated in Santa Cruz department over the past decade. However, these programs have been constrained by administrative barriers and inadequate surgical facilities. One program implanted an estimated 100 pacemakers yearly from 2005 to 2010, charging patients on a sliding scale, but has performed few procedures since 2010 because a crucial piece of surgical equipment stopped functioning, and neither the organization nor the hospital can afford to replace it (Puente de Solidaridad, http://www.puentesol.org/programas.html). Another program provided 30–40 free pacemakers yearly, with patients paying approximately US$800 each for the procedure, but the program was halted in 2012 by barriers to importation of donated pacemakers (Heartbeat International, https://www.heartbeatsaveslives.org/pacemaker-program/3/1/0/page.htm). A third non-profit program functioned in a similar manner, but stopped offering services for unspecified reasons (Niño Feliz, http://www.fnf.org.bo/index.php?option=com_content&view=article&id=60%3Amarcapasos&catid=37%3Amarcapasos&Itemid=2). Thus, even when all three programs were functioning, only 170–200 low-cost pacemakers were available annually in the Santa Cruz region, representing only 10% of the 1,800 patients estimated to need pacemakers.

One promising solution to inadequate device availability is reused pacemakers. Over 235,000 pacemakers are implanted in the United States every year. Many patients undergo upgrades to implantable cardioverter-defibrillators, and pacemakers often outlive their recipients [15]. Thus, thousands of pacemakers with significant battery life are explanted and returned to the manufacturers every year, or are buried or discarded by the funeral industry. Observational studies have demonstrated that reused devices are safe when sterilized appropriately [15], [16]. However, challenges exist to large-scale employment of reused pacemakers to fill the gap between availability and need, including regulatory constraints and barriers in import and export. Understandably, in the absence of a rigorous prospective study, health authorities question the safety of reused pacemakers. Currently, Food and Drug Administration (FDA) approval for a clinical trial of their safety and efficacy is being sought by the University of Michigan project My Heart Your Heart; favorable results will facilitate program scale-up. Strict criteria for sterilization and follow-up are essential. Patients need device interrogations every six months, a daunting challenge for patients who live in remote areas and lack funds for transportation. Even with a donated pacemaker, the cost of leads (which cannot be reused) and implantation are beyond the resources of most patients in southern Bolivia. A collaborative effort is required by pacemaker donation programs, US centers of excellence, local implantation centers, and the governments of Chagas disease-endemic countries to address this unmet need. With the necessary cooperation and infrastructure, pacemaker reuse programs have the potential to offer thousands of low-cost, lifesaving devices to impoverished patients with advancing Chagas cardiomyopathy.
